# High-Temperature Optoelectronic Transport Behavior of n-TiO_2_ Nanoball–Stick/p-Lightly Boron-Doped Diamond Heterojunction

**DOI:** 10.3390/ma18020303

**Published:** 2025-01-10

**Authors:** Shunhao Ge, Dandan Sang, Changxing Li, Yarong Shi, Cong Wang, Chunshuai Yu, Guangyu Wang, Hongzhu Xi, Qinglin Wang

**Affiliations:** 1 Shandong Key Laboratory of Optical Communication Science and Technology, School of Physics Science and Information Technology, Liaocheng University, Liaocheng 252000, China; gsh113026@163.com (S.G.); a17616235807@163.com (C.L.); syr2577974181@163.com (Y.S.); 13774956200@163.com (C.Y.); wangguangyu@lcu.edu.cn (G.W.); wangqinglin@lcu.edu.cn (Q.W.); 2 College of Mathematics and Physics, Beijing University of Chemical Technology, Beijing 100029, China; wangcongphysics@mail.buct.edu.cn; 3 Anhui Huadong Photoelectric Technology Research Institute Co., Ltd., Wuhu 241002, China; hdgdthz@163.com

**Keywords:** N-TiO_2_ NBSs, p-lightly boron-doped diamond, optoelectronic transport behavior, high temperature, backward diode

## Abstract

The n-TiO_2_ nanoballs–sticks (TiO_2_ NBSs) were successfully deposited on p-lightly boron-doped diamond (LBDD) substrates by the hydrothermal method. The temperature-dependent optoelectronic properties and carrier transport behavior of the n-TiO_2_ NBS/p-LBDD heterojunction were investigated. The photoluminescence (PL) of the heterojunction detected four distinct emission peaks at 402 nm, 410 nm, 429 nm, and 456 nm that have the potential to be applied in white-green light-emitting devices. The results of the *I-V* characteristic of the heterojunction exhibited excellent rectification characteristics and good thermal stability at all temperatures (RT-200 °C). The forward bias current increases gradually with the increase in external temperature. The temperature of 150 °C is ideal for the heterojunction to exhibit the best electrical performance with minimum turn-on voltage (0.4 V), the highest forward bias current (0.295 A ± 0.103 mA), and the largest rectification ratio (16.39 ± 0.005). It is transformed into a backward diode at 200 °C, which is attributed to a large number of carriers tunneling from the valence band (VB) of TiO_2_ to the conduction band (CB) of LBDD, forming an obvious reverse rectification effect. The carrier tunneling mechanism at different temperatures and voltages is analyzed in detail based on the schematic energy band structure and semiconductor theoretical model.

## 1. Introduction

Titanium dioxide (TiO_2_) possesses remarkable photoelectric properties and holds immense potential for various applications with its substantial band gap of 3.2 eV and a typical intrinsic n-type semiconductor material [1]. It exhibits excellent light absorption and photoelectric conversion efficiency, rendering it an optimal material for optoelectronic devices [2], including photodetectors, light-emitting diodes (LEDs), storage applications, and field-effect transistors (FETs) [3]. In the present study, nanostructured TiO_2_ was successfully deposited on various materials. Heris S. et al. specifically synthesized TiO_2_/ZnO nanoparticle hybrid materials, which significantly enhanced the photocatalytic degradation of tetracycline in wastewater under ultraviolet irradiation [4]. This improvement was primarily attributed to the superior electron–hole pair separation and a broader photo response range of the TiO_2_/ZnO heterostructures. The successful creation of this structure underscores the feasibility of titanium dioxide and its effective integration in heterogeneous architectures. Further, Jubu PR et al. developed a heterojunction structure comprising a TiO_2_ nanotube film decorated with β-Ga_2_O_3_ nanoparticles, markedly enhancing the photocatalytic efficiency of water decomposition [5]. The sol–gel method was employed to uniformly deposit β-Ga_2_O_3_ nanoparticles on TiO_2_ nanotubes, achieving outstanding electron–hole pair separation. The morphology and structure of the composite were verified through comprehensive characterization techniques, thus confirming the feasibility and effectiveness of titanium dioxide in constructing an efficient photocatalytic system. This development of heterogeneous structures provides robust material support for solar energy-driven water decomposition, showcasing its potential in sustainable energy technologies. Additionally, Pratyay A. A. et al. successfully engineered a heterostructure of TiO_2_ combined with AlAs for fabricating a double-gate structure MOSFET (DGJLMOSFET) [6]. Detailed analysis of the impact of gate oxide material type and thickness on device performance confirmed the suitability of TiO_2_ for use in advanced semiconductor devices. Particularly, in the heterostructure of TiO_2_ and AlAs, significant improvements were observed in electrical performance, including enhanced carrier mobility and reduced leakage current, highlighting its potential for high-performance electronic applications and the creation of pn heterojunctions with excellent properties. However, these heterojunctions present a challenge in maintaining stability and efficiency in extreme environments, particularly at elevated temperatures. In contrast, diamond exhibits unique physical properties, including exceptionally high thermal conductivity, superior hardness, and remarkable resistance to chemical and thermal degradation. These characteristics make diamond an excellent candidate for heat dissipation in high-temperature and high-power optical and electronic devices [7]. Combining TiO_2_ with diamond could potentially overcome the limitations of existing heterojunctions and enable the development of advanced optoelectronic devices that operate reliably in harsh environments. Despite the promising prospects of TiO_2_/diamond heterojunctions, there remains a significant knowledge gap in understanding their photoelectric transmission behavior under extreme conditions, especially at elevated temperatures [8]. While existing studies have investigated the properties of TiO_2_-based heterojunctions on other substrates, systematic exploration of their behavior when combined with diamond under varying thermal conditions is limited.

To address this gap, our team has conducted prior research on TiO_2_ nanorod clusters deposited on boron-doped diamond (BDD) thin films, laying a solid foundation for this study [9]. In this research, we focus on the temperature-dependent electron transport behavior of TiO_2_ nanoballs–sticks (NBSs) combined with LBDD thin films. From room temperature (25 °C) to 150 °C, the heterojunction demonstrates excellent rectification characteristics, achieving a maximum rectification ratio at 150 °C. At 200 °C, the heterojunction transitions to a reverse diode. Using a theoretical semiconductor model and corresponding band structure analysis, we elucidate the mechanisms driving these temperature-induced changes. This work provides new insights into the development of semiconductor optoelectronic devices capable of withstanding high-temperature and extreme environmental conditions.

## 2. Materials and Methods

In this study, the p-type diamond film used is BDD film, and its boron source is liquid trimethyl borate ((CH_3_O)_3_B). Boron-doped diamond films were prepared on p-type silicon wafers by the hot filament chemical vapor deposition (HFCVD) method in a reaction device filled with hydrogen (H_2_) and methane (CH_4_) gases. More detailed information is provided in the Supplementary Materials. Table S1 provides the key process parameters for the growth of boron-doped diamond film in this experiment. Concurrently, TiO_2_ NBSs were synthesized via a hydrothermal synthesis technique. The process entailed the placement of the p-LBDD film substrates within a reactor containing a reaction solution comprising three-quarters of the reactor volume. The precise composition of the solution was 0.2 mol/L TiCl_3_ and 3.6 mol/L NaCl [10]. Subsequently, the reactor was sealed, and the temperature was increased to 180 °C. The reaction temperature was maintained for eight hours to allow the desired chemical transformations to occur. Upon completion of the reaction period, the sample within the reactor was rinsed with ultrapure water in order to eliminate any residual reactants or by-products. Subsequently, the samples were air-dried under ambient conditions in preparation for subsequent characterization and analysis. However, because there may be a small temperature gradient inside the reactor, this can lead to differences in local deposition thickness. In addition, although the sample is cleaned with ultrapure water several times after the reaction to remove the residue, trace impurities in the reaction solution may form a defect state at the interface, which may have a slight impact on the photoelectrical properties. In subsequent studies, these uncertainties will be reduced by introducing more accurate temperature control equipment and reactor design optimization.

In this study, the Thermo Fisher Scientific FIB-SEM GX4 scanning electron microscope (model: 10.16.0.97, Waltham, MA, USA) was primarily used to observe the surface morphology. Operating with an accelerating voltage of 15.00 kV, a working distance of 4.1 mm, a probe current of 86 pA, an aperture size of 45 μm, and a magnification range of 10,000× to 100,000×, the SEM provided high-resolution images for analyzing the surface structure and uniformity of the samples. In addition, the Thermo Fisher Scientific FIB-SEM GX4 system is equipped with an energy-dispersive X-ray spectroscopy (EDS) detector to analyze the elemental composition and distribution of heterojunction films. EDS measurements are performed at an accelerated voltage of 15.00 kV and allow for accurate identification and mapping of titanium (Ti), boron (B), and carbon (C) elements, providing critical information on boron doping uniformity and overall composition uniformity of the film. X-ray diffraction (XRD) measurements were performed using a Bruker D8 ADVANCE diffractometer (Billerica, MA, USA). The instrument was operated with a scanning speed of 5°/min over a 2θ range of 5° to 80°, allowing for comprehensive acquisition of the XRD patterns to analyze the crystal structure and phase composition of the sample. Raman spectroscopy was performed using a Renishaw inVia micro-Raman spectrometer (Wotton-under-Edge, UK) with a 532 nm excitation laser source to analyze the structural and vibrational properties of the heterojunction. Fluorescence spectroscopy was conducted using an FLS920 fluorescence spectrometer (Edinburgh Instruments, Edinburgh, UK) with an excitation wavelength of 365 nm, enabling the analysis of the optical properties of the heterojunction. The carrier concentration of the diamond layer was measured using a low-temperature high-resistance transport system (ET9110-HS, Beijing, China), providing precise data on the electrical properties of the heterojunction. The *I-V* properties of the heterojunctions were measured using a Keithley 2400 SourceMeter (Cleveland, OH, USA), enabling accurate characterization of the electrical performance under various voltage conditions.

## 3. Results and Discussion

**Figure 1:**
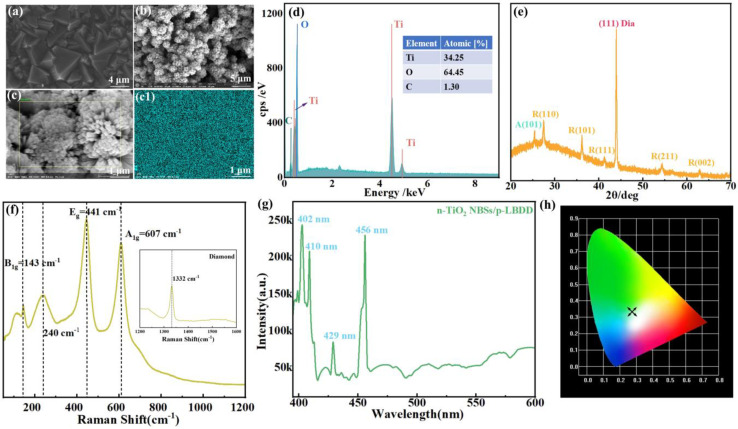
(**a**) SEM image of LBDD film. SEM image of TiO_2_ NBSs under low (**b**) and high (**c**) magnification. EDS mapping of Ti (**c1**) element corresponding to EDS images of TiO_2_ NBSs. (**d**) EDS spectra of n-TiO_2_ NBS/p-LBDD heterojunction; inset figure shows specific analysis of Ti, C, and O elements of n-TiO_2_ NBS/p-LBDD heterojunction. (**e**) XRD pattern of n-TiO_2_ NBS/p-LBDD heterojunction. (**f**) Raman spectra of n-TiO_2_ NBS/p-LBDD heterojunction; inset figure shows detected p-LBDD Raman peaks. (**g**) PL measurements of n-TiO_2_ NBS/p-LBDD heterojunctions at RT at 365 nm excitation wavelength. (**h**) CIE chromaticity map converted from test data.

As shown in Figure 1a, SEM images provide a detailed examination of the surface features of the p-LBDD film and TiO_2_ NBSs. The diamond film consists of small pyramidal diamond particles with sizes ranging from 1 µm to 3 µm. Supplementary Materials Figure S2 shows SEM images of a pure Si substrate and a Si substrate covered with diamond film from different perspectives_._ TiO_2_ NBSs were densely deposited on the surface of the LBDD film in an ordered ball structure consisting of tetrahedral stick shapes (i.e., ball–stick structure) with uniform distribution. The pore space between the balls and sticks is small, and the density of the prepared TiO_2_ NBSs is high (as shown in Figure 1b,c). However, due to possible local deposit thickness differences, this can lead to slightly different local current densities in some areas of the electrical test. However, repeated test results show that the overall performance of the sample is stable, indicating that the influence of deposition uniformity on the overall performance is limited. In order to analyze the elemental types and contents of the heterojunction microstrip components, the elemental surface distribution of each element of the heterojunction was investigated. Notably, the surface distribution of Ti shows high purity within the heterojunction interface (as shown in Figure 1(c1)), and the distributions of C and O are elaborated in the Supplementary Materials. The graphical measurements further confirm the uniform and tight distribution of Ti and O elements within the analyzed area in the selected region, while the distribution of C is sparser, indicating the effective coverage of the LBDD surface by TiO_2_ NBSs [7,8]. The quantitative decomposition of the normalized mass of C, O, and Ti elements, quantified as 0.58%, 38.38%, and 61.04%, respectively, is given in Figure 1d [11]. These values are consistent with the observed distribution pattern. In addition, the atomic ratio data show a Ti-O ratio of about 1:2, indicating the successful synthesis of high-purity n-TiO_2_ NBSs within a heterojunction structure.

X-ray diffraction (XRD) spectra obtained from arrays of TiO_2_ NBSs grown on LBDD substrates reveal the crystal structure of the material, as shown in Figure 1e. The analysis shows that the reflection peaks at 2θ angles of (27.46°), (36.16°), (41.24°), (54.34°), and (62.92°) correspond to the (110), (101), (111), (211), and (002) crystal planes of the TiO_2_ rutile phase, respectively. This peak pattern is consistent with the peak pattern corresponding to rutile TiO_2_ in JCPDS standard card #21-1276 [12]. Of particular importance is the pronounced peak observed at 2θ = 27.46°, indicating preferential growth of nanorods along the (110) direction, which is perpendicular to this crystallographic plane. This alignment elucidates the specific structural features and orientation of TiO_2_ NBSs on the substrate surface. In addition, another diffraction peak found at 2θ = 43.98° corresponds to the (111) crystallographic plane of diamond [13,14]. XRD analysis confirmed that the rutile phase dominates in the synthesized TiO_2_ NBSs. However, a weak peak associated with the anatase phase was also observed at 25.43°, corresponding to the (101) crystallographic plane of TiO_2_. This suggests that dynamic interactions and potential phase transitions occur at the TiO_2_–diamond interface during the preparation process. Figure 1f shows the Raman spectra of the TiO_2_ NBS/p-LBDD heterojunction. The rutile-type structure is quadrangular with two TiO_2_ units D^14^_4h_ (P4_2_/mnm) per cell and spatial group, yielding four Raman activity modes (1A_1g_ + 1B_1g_ + 1B_2g_ + 1E_g_) that consist of oxygen motions relative to a fixed center Ti ion, or vertically to the c-axis (A_1g_, B_1g_, and B_2g_ modes) or along the c-axis (E_g_ mode). The Raman spectrum of standard rutile TiO_2_ shows four first-order bands in the range of 100~900 cm^−1^ for B_1g_ (143 cm^−1^), E_g_ (447 cm^−1^), A_1g_ (612 cm^−1^), and B_2g_ (826 cm^−1^) [15]. In addition, second-order scattering features are also present, most notably at 240 cm^−1^. The prepared TiO_2_ NBSs are dominated by the E_g_ and A_1g_ modes as well as the second-order modes at 240 cm^−1^, while the B_1g_ and B_2g_ modes are very weak or absent [16]. In the E_g_ and A_1g_ modes, the magnitude of the Raman spectra changes significantly: the Raman frequency redshift increases and the peak intensity decreases. These variations are in agreement with the phonon confinement effect in one-dimensional rod-like nanocrystals [17]. Due to dimensional constraints perpendicular to the rod axis direction (a and b axes), the phonon confinement effect is more pronounced in the in-plane A_1g_ mode. Inelastic neutron scattering measurements and theoretical modeling show that the A_1g_ mode has flat branches along the (110) direction, located within the Ti-O stretching plane, where oxygen vibrations dominate. This phonon dispersion aligns with the observed redshift and the reduced intensity of the A_1g_ mode’s peaks [18,19]. It is also worth noting that a distinct peak can be observed at 1332 cm^−1^ as shown in the inset of Figure 1f, which corresponds to the strongest Raman peak of diamond, known as the “Diamond peak”, which corresponds to the C-C vibrational mode [20,21].

We observed the luminescence characteristics of the n-TiO_2_ NBS/p-LBDD heterojunction under 365 nm wavelength excitation (shown in Figure 1g). The results show that the structure exhibits four characteristic emission peaks at 402 nm, 410 nm, 429 nm, and 456 nm. Among them, the main emission peaks at 402 nm and 410 nm are similar to the band gap energy of rutile TiO_2_ crystals (3.20 eV, ca. 415 nm), which may originate from indirectly allowed leaps [22,23]. The weaker emission peaks appearing at 429 nm are in agreement with the rutile-phase TiO_2_, which is caused by free exciton complexation. The distinctive emission peaks at 456 nm fall in the range of the typical emission of the rutile phase (447–457 nm), which is usually considered to be due to a double electron capture defect center in the oxygen vacancy (f-center) [24,25,26]. XRD and Raman spectroscopy results show that TiO_2_ NBSs have a high purity rutile phase, but some defect-related emission peaks are observed in the photoluminescence characteristics, which may be related to the defect state caused by impurities or oxygen vacancies at the interface of TiO_2_ and LBDD. These defects may have an impact on carrier transport performance, and the deposition process will be further optimized in the future to reduce the introduction of impurities [27]. Based on the luminescence test data, we plotted a CIE chromaticity diagram (shown in Figure 1h). The chromaticity coordinates (0.2729, 0.3333) are located in the white-green region, which suggests that n-TiO_2_ NBS/p-LBDD heterojunctions may have potential applications in white-green light-emitting devices.

**Figure 2:**
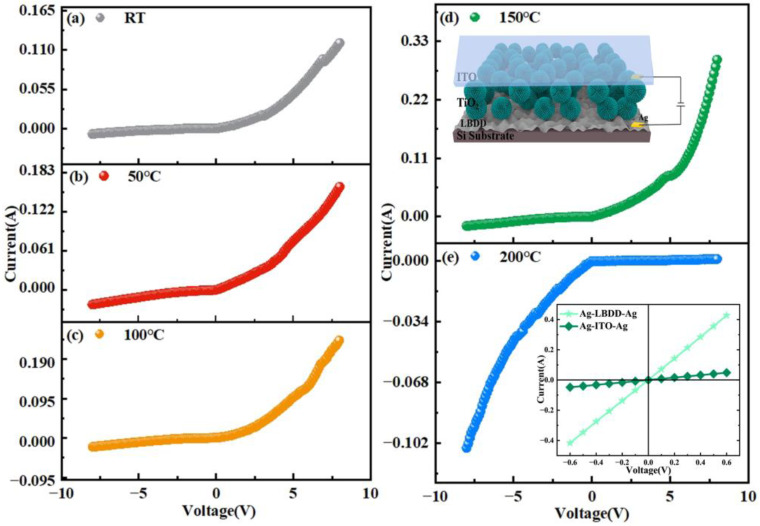
The *I-V* characteristic curves of n-TiO_2_ NBS/p-LBDD heterojunctions at various temperatures ranging from RT to 200 °C. (**a**–**e**) is the *I-V* characteristic curve of the heterojunction at RT, 50, 100, 150 and 200 °C, respectively. The top inset illustrates the schematic structure of the n-TiO_2_ NBS/p-LBDD heterojunctions, and the bottom inset shows the ohmic contact tests of Ag/ITO/Ag and Ag/LBDD/Ag.

Figure 2 (top inset) displays the schematic structure view of n-TiO_2_ NBS/p-LBDD heterojunctions. The TiO_2_ NBSs are contacted with the conductive side of transparent indium tin oxide (ITO) glass and fixed with cyanoacrylate adhesive to avoid direct contact between the conductive copper wires and TiO_2_, and the LBDD at the bottom of the contact will cause a short circuit, which can be avoided by using ITO as a dielectric. The conductive copper wire and the ITO are connected with silver paste, respectively, to make the heterojunction anode and cathode [10]. The linear *I-V* characteristics between the ITO/Ag and LBDD/Ag contacts show a linear relationship of an ohmic contact (Figure 2, bottom inset). TiO_2_-ITO also has ohmic contact characteristics due to the close work function. According to the Hall effect, the p-LBDD mobility is 27.5 cm^2^ V^−1^ s^−1^, the resistivity is 32.2 Ω cm, and the carrier concentration is 2.3 × 10^17^ cm^−3^.

The *I-V* characteristic curves of n-TiO_2_ NBS/p-LBDD heterojunctions were tested at temperatures ranging from 25 to 200 °C. It is observed that the bias current gradually increases under the same applied bias voltage as the temperature increases (Figure 2). At RT, the bias current of the n-TiO_2_ NBS/p-LBDD heterojunctions is measured to be 0.120 A ± 0.042 mA at 8 V (Figure 2a). However, the maximum current reaches 0.295 A ± 0.103 mA at 150 °C under a bias voltage of 8 V as shown in Figure 2d (specific data are listed in Table 1). The heterojunction shows classic pn junction diode behavior and exhibits rectification characteristics of 15 ± 0.005, 6.96 ± 0.002, 10.73 ± 0.006, and 16.39 ± 0.005 for all curves at ±8 V at 25 °C, 50 °C, 100 °C, and 150 °C, respectively. The rectification ratio’s variability across temperatures is primarily attributed to the temperature-dependent transport mechanisms. At lower temperatures, the rectification ratio is limited by thermal carrier injection and the interfacial barrier height. As the temperature increases to 150 °C, thermal excitation reduces the effective barrier height, leading to enhanced forward current and suppressed reverse current, which explains the observed peak rectification ratio at this temperature [19]. At 200 °C, the reverse current increases sharply due to the dominance of Fowler–Nordheim (F-N) tunneling, which becomes more significant at high temperatures [28]. This transition results in an apparent drop in the forward-to-reverse current ratio compared to 150 °C, consistent with the energy band structure’s thermal evolution. Since the n-TiO_2_ NBS/p-LBDD heterojunction has the lowest turn-on voltage (0.4 V), the highest forward bias current (0.295 A ± 0.103 mA), and the largest rectification ratio (16.39 ± 0.005) at 150 °C, the 150 °C temperature is thus considered to be the ideal temperature at which the heterojunction exhibits its best performance. It is noted that, at 200 °C, the heterojunction transforms into a backward diode with a reverse conduction voltage of 0.5 V and a rectification ratio of 105 ± 0.036 (Figure 2e). At high temperatures under reverse voltage, the thermally excited carrier concentration in the heterojunction increases significantly. This leads to a large number of carriers tunneling from the valence band of TiO_2_ to the conduction band of LBDD, resulting in a pronounced reverse rectification effect [29]. The fabricated n-TiO_2_ NBS/p-LBDD heterojunctions will be widely utilized in the device applications of reverse currents that need to be suppressed such as protection and control circuits for electronic devices. In addition, compared to ZnO/diamond and MoS_2_/diamond heterojunctions, the n-TiO_2_ NBS/p-LBDD structure exhibits improved electrical performance at high temperatures. The ZnO/diamond heterojunction shows significant reverse current leakage at 150 °C, limiting its rectification ratio to below 10 [30], while the MoS_2_/diamond heterojunction suffers from interface degradation beyond 200 °C [29]. In contrast, the n-TiO_2_ NBS/p-LBDD heterojunction maintains stable performance across this temperature range.

**Figure 3:**
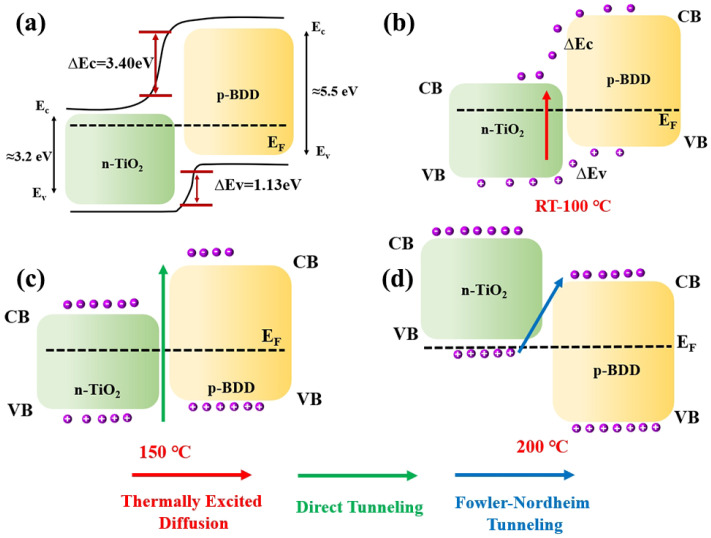
Energy band structure diagrams of n-TiO_2_ NBS/p-LBDD heterojunctions at different temperatures (RT-200 °C) based on the Anderson model. (**a**) is the band structure model of the heterojunction, and (**b**–**d**) are the band structure diagrams at low, medium and high temperatures respectively. Labels indicate the Fermi level (*E*_*F*_) and tunneling paths. At low temperatures, carrier transport is dominated by thermally excited diffusion and direct tunneling, while at high temperatures, F-N tunneling becomes significant.

The energy band structure diagram of the n-TiO_2_ NBS/p-LBDD heterojunction at different temperatures was constructed based on the Anderson model, as shown in Figure 3. The Anderson model is particularly well suited for describing semiconductor heterojunctions with large band offsets, as in the case of the n-TiO_2_ NBS/p-LBDD system. The large CB offset (ΔEc = 3.4 eV) and the smaller VB offset (ΔEv = 1.13 eV) in this heterojunction are effectively captured by the Anderson model [31], which considers the alignment of energy bands at the interface. The model helps to explain the dominant injection current mechanism in the heterojunction, which is primarily governed by the injection of holes into the VB [9]. Alternative models, such as the Schottky [32] or Mott–Schottky [33] models, were also considered, but they do not provide the same level of detail for this specific system, particularly in terms of the thermal behavior and carrier injection mechanisms under varying temperatures. At RT, holes and electrons are mainly transported by free diffusion due to the large barrier of potential. Meanwhile, a small number of holes are also able to pass from the VB of p-LBDD into the CB of n-TiO_2_ NBSs. As the temperature increases, the Fermi levels of the two materials gradually move to the mid-band gap position. This convergence is due to the thermal excitation of charge carriers resulting in an increased probability of hole transport from the VB of p-LBDD to the CB of the n-TiO_2_ and leading to an increased current of the n-TiO_2_ NBS/p-LBDD heterojunction (Figure 3b). The gradual contraction of the interfacial barrier and the decrease in the null energy state lead to a gradual decrease in the tunneling current as the temperature increases [34]. As the voltage increases further, natural diffusion currents and overcurrent states predominate and exhibit larger currents. When the temperature reaches 150 °C, the Fermi level of the two materials moves smoothly to the middle line of the band gap, further eliminating the interface barrier. Moreover, more hot carriers are excited and transmitted on the lower interface barrier [35] (Figure 3c), resulting in a further increase in current at high temperatures. When the temperature rises to 200 °C, the density of the thermal excited intrinsic carrier is further enhanced and increases the reverse current. Moreover, the Fermi level of TiO_2_ enters below the VB; the holes in the VB of TiO_2_ NBSs are more likely to tunnel into the CB of LBDD, forming a reverse tunneling current [36] (Figure 3d) and exhibiting the reverse rectification characteristic of a backward diode.

**Figure 4:**
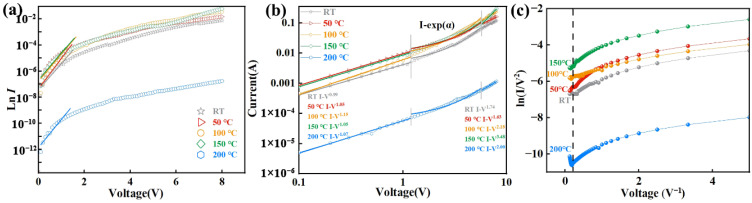
(**a**) The ln *I-V* plots of n-TiO_2_ NBS/p-LBDD heterojunctions at different temperatures. (**b**) Log *I*–log *V* plots of n-TiO_2_ NBS/p-LBDD heterojunctions at different temperatures. (**c**) ln (1/*V*^2^)-1/*V* diagram of n-TiO_2_ NBS/p-LBDD heterojunction at various temperatures.

The current transport mechanism of the n-TiO_2_ NBS/p-LBDD heterojunction at different temperatures is explored through the ln *I-V* characteristic curves [37,38] (Figure 4a). The ideal factor (*n*) is fitted by the Shockley diode Equation (1):

(1) $$I=I_{s}\left[\exp\left(\frac{qV}{nkT}\right)-1\right],$$

where *q* is the electron charge, *T* is the absolute temperature, *k* is the Boltzmann constant, $I_{s}$ is the reverse saturation current, and *V* is the applied voltage. The *n* of the heterojunction at each temperature is estimated to be 19.23 ± 0.006 (RT), 18.40 ± 0.006 (50 °C), 16.43 ± 0.006 (100 °C), 18.40 ± 0.006 (150 °C), and 17.72 ± 0.006 (200 °C), which are remarkably higher than that of the ideal diode *n* (2) [39]. The high *n* value of the heterojunction is caused by the presence of defects or impurities on the heterojunction surface (lattice mismatches and dislocations) [40]. The high *n* values can be attributed to the presence of interface trap states and tunneling effects. The lattice mismatch and defects at the n-TiO_2_ NBS/p-LBDD interface contribute to the formation of trap states, which capture and release charge carriers, affecting their injection and transport. Additionally, direct tunneling and F-N tunneling through the interfacial potential barrier further influence the current transport mechanism [41], especially at low bias voltages. At higher bias voltages, space charge accumulation may dominate the charge carrier transport, leading to deviations from the ideal diode behavior and higher *n* values [18,42]. This effect is more pronounced at elevated temperatures due to increased thermal excitation and defect-assisted carrier injection. There exist a number of energy levels or interface states that affect the carrier transport and injection further affects the *n* value. Although the *n* value obtained is relatively high, it is basically in line with the ideal factor range (17.30) of TiO_2_ heterojunctions reported previously [43].

To gain a deeper understanding of the current transport behavior of the n-TiO_2_ NBS/p-LBDD heterojunctions, the log *I*–log *V* curves for the heterojunction are plotted under different temperatures. According to the applied voltage, the curve is divided into three different regions as shown in Figure 4b. At lower voltages (region I), the current is mainly contributed by thermally excited carriers. According to thermal excitation theory, the density of the carrier is proportional to the exponential relationship $I\propto \exp\left(qV/kT\right)$ and the logarithmic transformation of the above equation yields $\log I=\left(qV/kT\right)+const$. It can be observed that the relationship between log *I* and *V* approximates a straight line in the lower voltage region. The slope of this line is *q*/*kT*, where *q* is the charge of an electron and *T* is the temperature. It indicates that the *I-V* characteristics of the heterojunction are directly dependent on the temperature variation. Through curve fitting analysis, the *I-V* characteristics of the heterojunction at different temperatures follow the power law relationships: *I-V*^0.99^, *I-V*^1.05^, *I-V*^1.15^, *I-V*^1.05^, and *I-V*^1.07^. It is suggested that the *I-V* relationship of the heterojunction is linear and adheres to Ohm’s law, where the current is proportional to the applied voltage [44]. It can be observed that the device exhibits nonlinear effects at a medium forward voltage (region II), at which the conductance characteristics follow $I\propto \exp\left(\alpha V\right)$ and conform to the wide band gap pn heterojunction to compound the recombination tunneling mechanism [45]. The fitted injection efficiencies *α* are 0.53 (RT), 0.46 (50 °C), 0.57 (100 °C), 0.46 (150 °C), and 0.44 (200 °C), which are within the range of ideal semiconductor junctions (0–1) [46]. In the high bias region III, the *I-V* plot followed the relationship of *I-V*^1.74^, *I-V*^1.63^, *I-V*^2.18^, *I-V*^3.48^, and *I-V*^2.0^. The increase in the index value to the standard value of 2 at all temperatures can be attributed to the space charge limiting current (SCLC) model at high voltages. This phenomenon occurs when charge carriers are confined by space charge accumulation within the device structure [28]. The exponent values gradually increase at high temperatures (RT-150 °C), mainly due to trap states from oxygen surface defects in the TiO_2_ NBSs. These defects capture thermally emitted carriers, facilitating the transfer of electrons into the diamond CB. This behavior aligns with the carrier injection analysis shown in the energy band diagrams (Figure 3b,c). When the temperature reaches 200 °C, the index becomes a standard value of 2 indicating that the fabricated TiO_2_ NBS/LBDD heterojunction is an ideal backward diode that obeys the standard trap-free SCLC transmission model.

To gain better insight to the interfacial carrier migration mechanism in depth, the current transport behavior of n-TiO_2_ NBS/p-LBDD heterojunctions can be explored by ln *(I*/*V*^2^*)* and 1/*V* curves using an F-N tunneling model [47] (Figure 4c).

(I) A large number of carriers will be excited when the external temperature increases; the energy provided by this thermal excitation enables the carriers to overcome the potential barrier and traverse the interface. The transmission mechanism can be elucidated by combining Equation (2):

(2) $$I=AA^{*}\exp[\frac{-(\emptyset_{b}-\sqrt{\frac{q^{3}V}{4\pi \varepsilon_{0}\varepsilon_{r}d}})}{KT}],$$

*d* represents the barrier height at the interface, *∅_b_* denotes the barrier height at *T* = 0 K, *ε_r_* represents the semiconductor dielectric constant, *A* represents the area of the heterojunction, *ε*_0_ denotes the vacuum dielectric constant, and A* represents Richardson’s constant. Based on Shockley’s theory and diffusion current, the formula describes the relationship between diode forward bias current and voltage and takes into account the influence of barrier height *∅_b_* and junction width on current through exponential terms. The ability of thermally excited carriers to overcome the interfacial barrier is affected by the external excitation temperature. In the case of n-TiO_2_ NBS/p-LBDD heterojunctions, the barrier height plays a crucial role in determining the thermal excitation required for carrier migration. As the temperature increases, the carriers gain more thermal energy and are thus able to overcome the higher potential barrier and promote current flow through the interface. The barrier height is proportional to the desired excitation temperature [48]. However, at lower temperatures, the thermal energy is not sufficient to overcome the potential barrier, and the carriers mainly utilize the tunneling mechanism to cross the interfacial potential barrier [49]. This result is in alignment with the analysis of energy band diagrams in the n-TiO_2_ NBS/p-LBDD heterojunction.

(II) The heterojunction carrier transport mechanism is mainly attributed to direct tunneling at low bias voltage, as shown in Equation (3) as follows:

(3) $$\ln\left(\frac{I}{V^{2}}\right)\propto \ln\left(\frac{1}{V}\right)-\frac{4\pi d\sqrt{2m\emptyset_{b}}}{h},$$

The given equation incorporating Planck’s constant (*h*) and the carrier effective mass (*m*) illustrates a linear relationship between ln(*I*/*V*^2^) and ln(1/*V*) at lower voltages, where the current is inversely proportional to the inverse of the voltage and the square root of the interfacial barrier. This relationship indicates that direct tunneling is a quantum mechanical effect that probabilistically transports carriers through the potential barrier.

(III) When the bias voltage is high, the carrier transport mechanism of the heterojunction is dominated by F-N tunneling, as shown in Equation (4):

(4) $$\ln\left(\frac{1}{V^{2}}\right)\propto -\frac{1}{V\left(\frac{8\pi d\sqrt{2m\emptyset_{b}^{3}}}{h}\right)},$$

The conduction mechanisms in the n-TiO_2_ NBS/p-LBDD heterojunction can be categorized into thermionic emission, direct tunneling, and F-N tunneling primarily dependent on temperature and voltage, respectively [41]. At lower temperatures (RT and 50 °C), a less pronounced inflection point appears on the *I-V* curve, indicating the presence of both direct and F-N tunneling [42]. At voltages higher than 3.2 V, the value 1/*V* exhibits a negative slope indicating the dominance of the F-N tunneling mechanism. At voltages lower than 3.2 V, the curve varies logarithmically with 1/*V* representing the influence of direct tunneling. It is worth noting that, at lower temperatures, due to the large barrier heights of the two materials despite the presence of complex tunneling, the carrier transport mechanism is mainly dependent on the natural diffusion current resulting in the inflection point being less pronounced. At higher temperatures (100 °C and 150 °C), the inflection point on the curve gradually diminishes as carriers gain higher thermal excitation energy to pass through the barrier. At 200 °C, an inflection point appears at *V* = 3.5 V in the reverse bias voltage region due to a band gap reduction. This observation indicates the presence of both direct tunneling and F-N tunneling effects that are consistent with the energy band diagram explanation [50]. With increasing applied bias voltage, the appearance of a large reverse current could be assigned to thermally activated carriers and enhanced generated currents [29]. The electrical transport mechanism at 200 °C above the n-TiO_2_ NBS/p-LBDD heterojunction supplied an effective physical principle for preparing efficient high-temperature backward diodes.

## 4. Conclusions

In conclusion, the rutile-phase n-TiO_2_ NBSs were deposited on p-LBDD by the hydrothermal method in order to form heterojunctions. The PL emission peaks observed at 402 nm, 410 nm, 429 nm, and 456 nm have the potential for application in white-green light-emitting devices. The *I-V* characteristic of the heterojunction displays excellent rectification behavior across a temperature range of RT-200 °C. The maximum rectification ratio and the minimum turn-on voltage were observed at 150 °C. At 200 °C, the material undergoes a transformation into a backward diode. The observed variation in the electrical properties of the heterojunction can be attributed to two main factors: the thermal excitation effect and the movement of the Fermi level. These factors lead to a change in carrier tunneling injection at high temperatures. The study shows that the n-TiO_2_ NBS/p-LBDD heterojunction has good rectification characteristics and thermal stability under the test conditions. These results highlight the potential of this heterojunction in applications requiring high heat resistance. While the results are promising, the study has certain limitations. The environmental conditions tested in this work (up to 200 °C) represent a limited range of potential applications, and additional experiments are needed to explore more extreme environments, such as high humidity or corrosive atmospheres.

## Figures and Tables

**Table 1:** Various electrical parameters of the n-TiO_2_ NBS/p-LBDD heterojunction under different temperatures.

Temperature (°C)	RT	50 °C	100 °C	150 °C	200 °C
Current at 8 V (A)	0.120 ± 0.042 mA	0.160 ± 0.056 mA	0.236 ± 0.082 mA	0.295 ± 0.103 mA	0.001 ± 0.0003 mA
Current at −8 V (A)	0.008 ± 0.002 mA	0.023 ± 0.008 mA	0.022 ± 0.007 mA	0.018 ± 0.006 mA	0.105 ± 0.036 mA
Rectification ratio	15 ± 0.005	6.96 ± 0.002	10.73 ± 0.003	16.39 ± 0.005	105 ± 0.036
Turn-on voltage (V)	0.8	0.4	0.6	0.4	0.5
Ideality factor *n*	19.23 ± 0.006	18.40 ± 0.006	16.43 ± 0.006	18.40 ± 0.006	17.72 ± 0.006

## Data Availability

The original contributions presented in the study are included in the article/Supplementary Materials, further inquiries can be directed to the corresponding author.

## Supplementary Materials

The following supporting information can be downloaded at: https://www.mdpi.com/article/10.3390/ma18020303/s1, Figure S1: EDS mapping of O(a) and C(b) element corresponding to EDS images of TiO_2_ NBSs; Figure S2: The SEM image shows (a) Si substrate, (b) obvious dividing line between LBDD nanostructures and Si, and (c) LDBB nanostructures are uniformly covered on Si substrate; Figure S3: The SEM image (a) shows a lightly boron-doped diamond film, (b) displays a heavily boron-doped diamond film; Table S1: Key process parameters of boron-doped diamond film growth in this experiment.
