# Supplementary material for: High-Temperature Optoelectronic Transport Behavior of n-TiO2 Nanoball–Stick/p-Lightly Boron-Doped Diamond Heterojunction
Source: Materials (Basel). 2025 Jan 10;18(2):303. doi: 10.3390/ma18020303 (PMC11766722; doi:10.3390/ma18020303)
Supplement: Supplementary file 1 [file materials-18-00303-s001.zip › materials-3406255-supplementary.pdf]

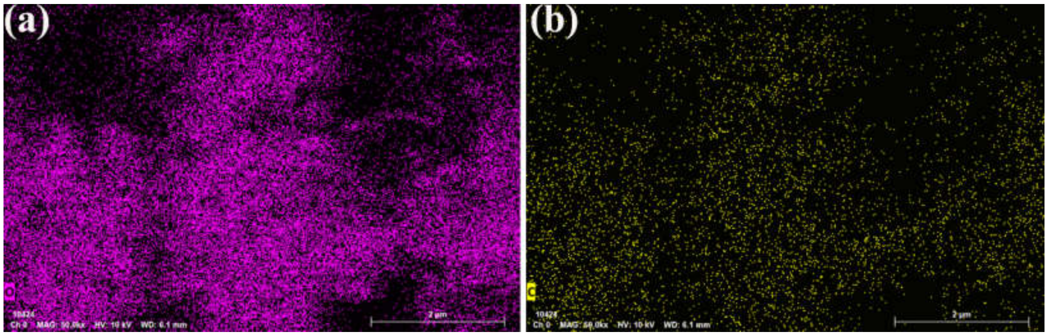

Figure S1 EDS mapping of O(a) and C(b) element corresponding to EDS images of TiO_2 NBSs.

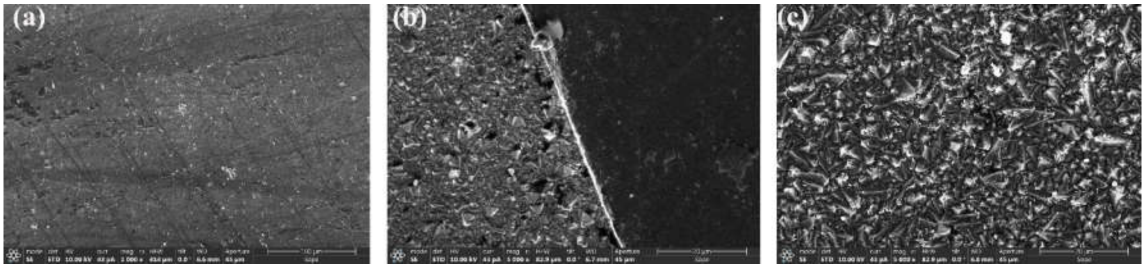

Figure S2 The SEM image shows (a) Si substrate, (b) obvious dividing line between LBDD nanostructures and Si, and (c) LDBB nanostructures are uniformly covered on Si substrate.

Detailed preparation technology of LBDD film

In this study, the p-type diamond thin films used were BDD thin films, with liquid trimethyl borate ($(CH_3O)_3B$) as the boron source. The BDD thin films were prepared on p-type silicon substrates via the hot filament chemical vapor deposition (HFCVD) method in a reaction chamber filled with hydrogen (H_2) and methane (CH_4). To ensure a high nucleation density, the silicon substrates underwent a series of pretreatment steps prior to deposition:

1. The substrate surfaces were polished with diamond powder to enhance nucleation density.

2.The polished substrates were ultrasonically cleaned in acetone for 20–30 minutes.

3.A secondary ultrasonic cleaning was performed in absolute ethanol for 5–10 minutes.

4.The cleaned substrates were dried using nitrogen gas (N_2) before being placed on the sample holder.

After substrate preparation, the chamber was evacuated, and CH_4 and H_2 gases were introduced for nucleation and growth on the silicon surface. The boron source (trimethyl borate) was introduced into the chamber via H_2. The boron doping level in the BDD thin films was effectively controlled by adjusting the H_2 flow rate. Specific reaction parameters for LBDD films are provided in Table

s1, while for heavily boron-doped films, the boron source flow rate was adjusted to 10 sccm. Hall effect measurements were conducted on the LBDD films, revealing that the BDD films exhibit p-type semiconductor behavior with a carrier concentration of 3.8×10^{17} cm^{-3}, mobility with 48.9 $cm^2/V\cdot s$ and resistivity with 0.33Ω ·cm.

Table S1 Key process parameters of boron-doped diamond film growth in this experiment

**Parameter**	**Experimental Condition**
Substrate material	p-type silicon wafer
Substrate thickness	0.5 mm
Working pressure	8.0 kPa
H_2 flow rate	400 sccm
CH_4 flow rate	5 sccm
Boron source flow rate	1 sccm
Deposition time	4.5 hours
Working temperature	900 °C

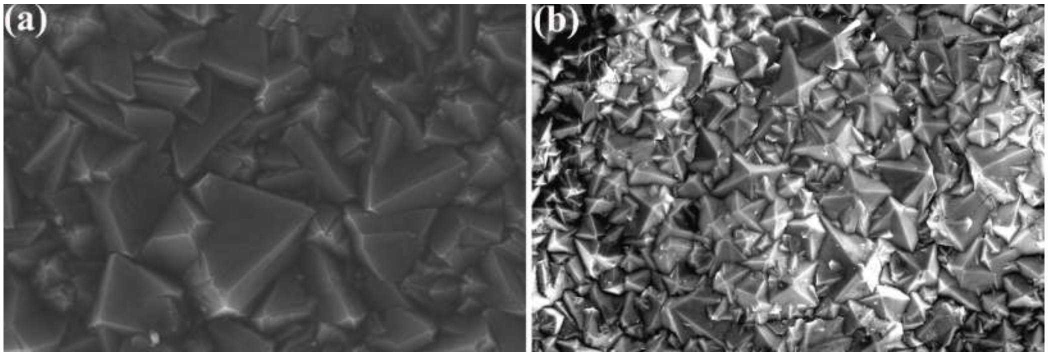

Figure S3 The SEM image (a) shows a lightly boron-doped diamond film, (b) displays a heavily boron-doped diamond film.

Lightly Doped Film (a): This film displays larger crystals with distinct, sharp edges. The crystals have sharp boundaries and a relatively smooth surface, with a regular arrangement in the crystal structure. Heavily Doped Film (b): In this film, the crystals are smaller and exhibit fewer regular shapes, with overlapping and twinning phenomena being quite common. The edges of the crystals are blurred, and the surface roughness is higher.
